# Three-Phase Serum Concentration Kinetics of FGF23 in a Clinical Model of Acute Myocardial Infarction

**DOI:** 10.3390/cells15080728

**Published:** 2026-04-20

**Authors:** Nora Strack, Praveen Gajawada, Christoph Liebetrau, Oliver Dörr, Till Keller, Yeong-Hoon Choi, Manfred Richter

**Affiliations:** 1 Kerckhoff Heart Center, Department of Heart Surgery, Campus Kerckhoff, Justus Liebig University Giessen, 61231 Bad Nauheim, Germany; nora.strack@googlemail.com (N.S.); y.choi@kerckhoff-klinik.de (Y.-H.C.); 2 Cardioangiological Center Bethanien, 60389 Frankfurt, Germany; c.liebetrau@ccb.de (C.L.); o.doerr@ccb.de (O.D.); 3 Kerckhoff Heart Center, Department of Cardiology, Campus Kerckhoff, Justus Liebig University Giessen, 61231 Bad Nauheim, Germany; till.keller@med.uni-giessen.de; 4 Department of Cardiology, Justus Liebig University of Giessen, 35392 Giessen, Germany; 5 German Center for Cardiovascular Research (DZHK), Partner Site Rhine-Main, 60325 Frankfurt am Main, Germany

**Keywords:** fibroblast growth factor 23, biomarker, acute myocardial infarction, TASH, STEMI, inflammation, heart failure, cardiomyocyte, Oncostatin M

## Abstract

**Highlights:**

**What are the main findings?**

- We report a novel, triphasic kinetic profile of circulating FGF23 within the first 24 h following acute myocardial injury in humans.
- FGF23 levels demonstrate an immediate peak at 30–60 min, followed by a significant transient decline at 4 h and a secondary rise by the 24-h mark.

**What is the implication of the main finding?**

- The rapid fluctuations suggest that FGF23 is a dynamic early responder to acute cardiac stress, rather than solely a marker of chronic systemic or mineral metabolism changes.
- This temporal characterization provides a precise clinical framework for future studies investigating the early biological functions and diagnostic potential of FGF23 in acute coronary syndromes.

**Abstract:**

Background: Fibroblast growth factor-23 (FGF23) is a key regulator of phosphate homeostasis and an emerging biomarker in cardiovascular disease. Emerging data suggest that FGF23 may also contribute to the pathophysiology of myocardial infarction (MI), but existing studies have largely focused on non-acute stages. To address this gap, we investigated early FGF23 regulation by characterizing serum concentration kinetics over the first 24 h following MI, using both a clinical MI model (TASH) and a cohort of patients with ST-elevation myocardial infarction (STEMI). Methods: Circulating FGF23 concentrations (cFGF23; RU/mL) were determined by C-terminal ELISA in patients with preserved renal function (eGFR > 30 mL/min/1.73 m^2^). TASH (transcoronary septal ablation) was carried out in patients with hypertrophic obstructive cardiomyopathy (*n* = 38). Venous serum samples were taken at baseline (pre-TASH) and at 30′, 60′, 2 h, 4 h and 24 h post-TASH. For the STEMI cohort (*n* = 18), serum was sampled immediately before and 3 h after coronary recanalization. All samples were processed using standardized procedures prior to analysis. Changes over time were assessed using the Friedman test with Bonferroni-corrected pairwise Wilcoxon comparisons. Results: FGF23 concentrations changed significantly over time after TASH (Friedman test, *p* < 0.000001, Kendall’s W = 0.518). Baseline FGF23 was 28.9 (19.4–71.0) RU/mL and increased significantly at 30′ (68.2 (36.2–178.7) RU/mL, adjusted *p* < 0.0001 **) after TASH. Concentrations remained elevated at 60′ (54.8 (31.6–118.3) RU/mL; adjusted *p* = 0.0019 *), returned to baseline at 2 h (30.9 (20–71.2) RU/mL; adjusted *p* = 1.0 vs. baseline) and decreased significantly below baseline at 4 h (24 (12.13–37.5) RU/mL, adjusted *p* = 0.0215 *). By 24 h, FGF23 had returned to baseline levels (28.8 (12.8–57.3) RU/mL; adjusted *p* = 1.0 vs. baseline). Although concentrations were numerically higher than at the 4 h nadir, this recovery did not reach statistical significance (adjusted *p* = 0.136 vs. 4 h). In STEMI patients, a non-significant decrease was observed from baseline (27 (15.5–35.75) RU/mL) to 3 h after recanalization (15.5 (6.75–34.25) RU/mL; *p* = 0.074, effect size r = 0.422). In an exploratory normalized analysis, the decline reached significance (*p* = 0.0241). Conclusions: The triphasic kinetics of circulating FGF23 in TASH patients—characterized by an early rise, transient undershoot, and a recovery toward baseline with a continuing upward trend—are consistent with a dynamic release-and-clearance pattern following myocardial injury. These findings are hypothesis-generating and warrant further investigation in larger cohorts with additional biomarkers to elucidate the source, regulation, and potential functional significance of FGF23 in the acute phase of myocardial infarction.

## 1. Introduction

Fibroblast growth factor 23 (FGF23), along with FGF19 and FGF21, constitutes the FGF19 subfamily of endocrine hormones that regulate mineral metabolism. Originally recognized as a biomarker for renal failure, FGF23 is now emerging as a biomarker in cardiac disease, with growing evidence linking this hormone to the development of various cardiac conditions [1,2,3]. Although FGF23 expression has been detected in the brain, thymus, liver, and heart, the primary source of circulating FGF23 is bone, owing to robust production by osteoblasts and osteoclasts [3,4,5].

However, recent findings indicate that FGF23 can also be produced by non-bone cells, including those in the heart, under certain conditions. The first clear demonstration came from cell culture experiments where cardiomyocytes and cardiac fibroblasts secreted high levels of FGF23 when stimulated with oncostatin M (OSM)—an interleukin-6 family cytokine that is abundantly active in acutely infarcted myocardium [3,6]. Subsequent studies confirmed FGF23 production by other cell types such as fibroblasts [7,8], endothelial cells [8], and macrophages [9]. Consistent with these findings, cardiomyocytes positive for FGF23 have been identified in patients with various cardiac diseases, including ischemic cardiomyopathy, coronary heart disease, dilated cardiomyopathy, and myocarditis [3,6,10]. Moreover, in animal models, elevated cardiac expression and circulating levels of FGF23 have been demonstrated in mice with myocarditis that progresses to dilated cardiomyopathy [6], as well as in rats and mice following acute myocardial infarction [7,11].

In clinical settings, changes in FGF23 levels have also been observed after myocardial infarction (MI). For instance, patients with infarction-related cardiogenic shock exhibit significantly higher serum FGF23 compared to those with stable coronary artery disease or non-shock MI [12]. Similarly, revascularized ST-elevation MI (STEMI) patients who undergo adverse left ventricular remodeling show increased circulating FGF23 levels [13]. Furthermore, dynamic variations in serum FGF23 during the first week post-infarction have been documented in STEMI patients following percutaneous coronary intervention (PCI) [14]. Notably, Takahashi et al. reported that STEMI patient FGF23 levels remain essentially unchanged from baseline to 2 days post-PCI, but then rise and stay elevated from 1 week up to 1 year after the infarction, a delayed yet prolonged FGF23 response that highlights important temporal dynamics and suggests different underlying mechanisms in the acute versus later phases of myocardial remodeling [14].

Despite these advances, no study to date has examined FGF23 levels in the very first hours after MI onset, the critical window for infarct marker detection and acute therapeutic intervention. This gap in knowledge is especially compelling given two key observations from our prior work. First, neutrophils massively infiltrate the infarcted myocardium within hours of injury and can rapidly release OSM from pre-formed granule stores. Second, OSM subsequently orchestrates the recruitment of OSM-secreting macrophages, which leads to local FGF23 expression and secretion by cardiac cells within hours [15]. These insights underscore the plausibility of an early surge in FGF23 following myocardial injury and reinforce the importance of investigating FGF23 immediately after infarction onset.

## 2. Methods

### 2.1. Patients and Treatment

This observational study used serum samples that had been prospectively collected at predefined time points as part of a biomarker research protocol. FGF23 measurements were performed retrospectively on stored samples. The study included two cohorts of patients. The treatment of patients with hypertrophic obstructive cardiomyopathy using transcoronary septal ablation (TASH) has been previously described in detail [16]. Briefly, TASH induces a controlled, localized myocardial infarction by injection of ethanol into a septal perforator artery, thereby serving as a clinical model for studying biomarker kinetics following acute myocardial injury.

For the TASH cohort (*n* = 38), patients were enrolled between November 2012 and July 2021 at Kerckhoff clinic. For the STEMI cohort (*n* = 18), patients presenting with ST-elevation myocardial infarction were enrolled between February 2012 and October 2013 at Kerckhoff clinic. All participants provided written informed consent. The study was approved by the local ethics committee (AZ 199/15).

Inclusion required signed informed consent. Exclusion criteria were: symptomatic carotid stenosis, prior disabling stroke, liver cirrhosis or chronic active hepatitis or severe hepatic dysfunction (transaminases > 3-fold elevated), uncontrolled diabetes mellitus (fasting glucose > 24 mmol/L), significant ongoing alcohol or drug abuse, concurrent participation in other studies, prior peripheral arterial surgery or angioplasty, severe renal impairment (creatinine clearance < 30 mL/min), malignancy within the preceding 5 years, and hepatitis B, hepatitis C, or HIV infection.

In the TASH cohort, venous serum samples were obtained at baseline (immediately before TASH) and at 30 min, 60 min, 2 h, 4 h, and 24 h after the procedure. In the STEMI cohort, serum was sampled at hospital admission (before recanalization) and 3 h after percutaneous coronary intervention (PCI). Baseline medications were recorded but not standardized given the observational nature of the study.

### 2.2. Laboratory Assessment

The ELISA that recognizes the C-terminal fragment of FGF23 was obtained from Immunotopics, Inc., San Clemente, CA, USA [6]. The ELISA assay used does not discriminate between full-length and fragmented FGF23, determining total FGF23 instead. According to the manufacturer’s information, 1 RU/mL corresponds to approximately 1.5 pg/mL. This assay was chosen because it captures both intact FGF23 and its C-terminal fragments, providing a measure of total circulating FGF23. However, it does not allow differentiation between biologically active intact FGF23 and inactive fragments, which should be considered when interpreting the results. This assay provides a sensitivity of 1.5 RU/mL and features a dynamic range extending up to approximately 445 RU/mL at 450 nm, and the ability to measure concentrations up to 1.400 RU/mL via secondary wavelength monitoring at 620 nm. To ensure analytical precision, all standards, controls, and patient samples were measured in duplicate, and the average absorbance was used for final concentration calculations. The assay’s technical rigor is further demonstrated by an intra-assay coefficient of variation (CV) ranging from 1.1% to 2.4% and an inter-assay CV ranging from 2.4% to 4.7%, ensuring robust results for this biomarker analysis.

Blood samples were collected in serum tubes, allowed to clot for 30 min, and then centrifuged at 3000 rpm. The supernatant was aliquoted and stored at −80 °C until analysis. There were no freeze–thaw cycles as all measured samples were thawed only once. All samples from the same patient were analyzed on the same plate to minimize batch effects. Laboratory personnel were blinded to the time point of each sample during analysis but were aware of cohort assignment (TASH vs. STEMI). No other clinical information was available to the analyst.

### 2.3. Statistical Analysis

All statistical analyses were performed using Python 3 with the SciPy and Pingouin libraries. Continuous variables are presented as median and interquartile range (IQR). Bootstrapped 95% confidence intervals (CIs) for paired median differences were calculated using 10,000 resamples.

A two-sided *p*-value ≤ 0.05 was considered statistically significant.

For the TASH cohort (*n* = 38), changes in C-terminal FGF23 concentrations across six time points (baseline, 30 min, 1 h, 2 h, 4 h, and 24 h) were analyzed using the Friedman Test, with Kendall’s W reported as effect size. Post hoc pairwise comparisons were performed using the Wilcoxon signed-rank test with Bonferroni correction for multiple testing (15 comparisons). Effect sizes for pairwise comparisons were calculated as r = Z/√n.

Both absolute FGF23 concentrations and baseline-normalized values (expressed as percentage of individual baseline) were analyzed. The normalized analysis was pre-specified as an exploratory sensitivity analysis to account for inter-individual variability.

For the STEMI cohort (*n* = 18), changes between baseline and 3 h were assessed using the Wilcoxon signed-rank test with effect size r. An exploratory analysis of baseline-normalized values was also performed.

In the TASH cohort, missing data were due to logistical reasons and were not related to patient characteristics or FGF23 levels. The Friedman test was conducted on complete cases (*n* = 31), while pairwise comparisons used all available paired observations to maximize statistical power.

## 3. Results

Table 1 summarizes the clinical characteristics of the hypertrophic obstructive cardiomyopathy (HOCM) patients who underwent therapeutic septal ablation (TASH). This cohort consisted of 38 patients (23 women and 15 men) with a mean age of 64.2 ± 11.7 years and preserved left ventricular ejection fraction (mean EF 64.2% ± 7.4%). Renal function was within age-appropriate normal limits (mean glomerular filtration rate 77.2 ± 20.5 mL/min; serum creatinine 0.9 ± 0.2 mg/dL), well above the exclusion threshold of 30 mL/min, indicating proper kidney function. The procedural details of septal ethanol ablation and the kinetics of standard cardiac biomarkers in this patient group (e.g., C-reactive protein, CK-MB, cardiac troponin T, myoglobin, interleukin-6) have been documented previously [16,17].

**Figure 1:**
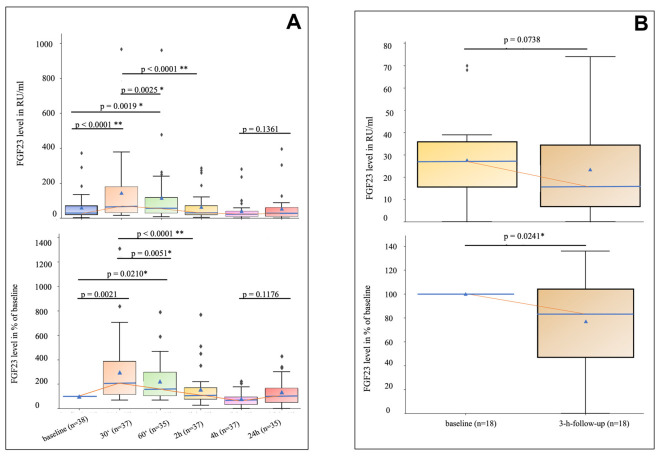
Time course of total FGF23 concentrations in serum of patients after TASH and in STEMI patients after recanalization. (**A**) FGF23 levels were measured before (baseline) and at 30 min, 60 min, 2 h, 4 h, and 24 h after TASH (baseline: *n* = 38; 30 min: *n* = 37; 1 h: *n* = 35; 2 h: *n* = 37; 4 h: *n* = 37; 24 h: *n* = 35). In the upper panel, FGF23 concentrations are shown in RU/mL; the lower panel shows values normalized to each patient’s baseline (baseline = 100%) as an exploratory sensitivity analysis to account for inter-individual variability. Note that the sample size (*n*) varies at different time points because some patients did not have blood samples collected at certain times. (**B**) FGF23 levels were measured at hospital admission (baseline) and 3 h after recanalization in STEMI patients (*n* = 18). The upper panel shows the absolute FGF23 concentration (RU/mL); the lower panel shows baseline-normalized values (exploratory analysis). Pairwise comparisons were performed using the Wilcoxon signed-rank test with Bonferroni correction for 15 comparisons (TASH) or without correction (STEMI, single comparison). * *p* < 0.05; ** *p* < 0.01 (Bonferroni-corrected). Boxes represent the interquartile range (25th to 75th percentile). The horizontal line within each box indicates the median, and the blue triangle denotes the mean. Whiskers extend to 1.5 times the interquartile range. Rhombuses represent individual patient data points.

FGF23 serum concentrations were measured at baseline (immediately before infarction induction) and at 30 min, 60 min, 2 h, 4 h, and 24 h after TASH (Figure 1A). Of 38 patients, 31 had complete measurements at all six time points (available observations per time point ranged from 35 to 38; missingness was due to logistical reasons and unrelated to patient outcomes). The Friedman test confirmed a significant change in FGF23 concentrations over time (χ^2^ = 80.332, *p* < 0.000001, Kendall’s W = 0.518, indicating a large effect). Post hoc pairwise comparisons were performed using the Wilcoxon signed-rank test with Bonferroni correction for 15 comparisons. Within 30 min of TASH, FGF23 levels rose sharply from a baseline median of 28.9 RU/mL (IQR 19.4–71.0) to 68.2 RU/mL (IQR 36.2–178.7), a ~2-fold increase [95% CI: 15.70 to 52.90] (adjusted *p* < 0.0001). By 2 h, FGF23 had returned to baseline levels (median 30.9 RU/mL, IQR 20.0–71.2; median difference vs. baseline +1.40 RU/mL [95% CI: −5.20 to 8.10], adjusted *p* = 1.0). By 4 h post-TASH, FGF23 reached a nadir of 24.0 RU/mL (IQR 12.1–37.5), significantly lower than the baseline (median difference −8.00 RU/mL [95% CI: −21.40 to −1.90], adjusted *p* = 0.022) and the 30-min peak (median difference −42.35 RU/mL [95% CI: −65.25 to −26.10], adjusted *p* < 0.0001). From this 4-h nadir, FGF23 showed a notable recovery, rising back to 28.8 RU/mL (IQR 12.8–57.2) by 24 h—a clear upward trend that is visually evident in Figure 1A (median difference +5.10 RU/mL [95% CI: −0.40 to 17.30], raw *p* = 0.009). Although this increase did not retain significance after Bonferroni correction for 15 comparisons (adjusted *p* = 0.14), FGF23 levels at 24 h had returned to baseline concentrations (24 h vs. baseline: adjusted *p* = 1.0). Notably, the normalized analysis showed a median of 102% of baseline at 24 h (IQR 51–162%), with a substantial proportion of patients exceeding their individual baseline values. Given that FGF23 was still rising between 4 and 24 h, it is conceivable that this upward trajectory may continue beyond the 24-h observation window, potentially reflecting a secondary increase in FGF23 following the initial release-and-decline phase. Overall, 11 of 15 pairwise comparisons remained statistically significant after Bonferroni correction.

As a pre-specified exploratory sensitivity analysis, FGF23 values were normalized to each patient’s own baseline (baseline = 100%) to account for substantial inter-individual variability in absolute concentrations (Figure 1A, lower panel). The Friedman test confirmed a significant effect of time on normalized values (χ^2^ = 73.006, *p* < 0.000001, Kendall’s W = 0.471). The temporal pattern was consistent with the absolute analysis, with 10 of 15 pairwise comparisons reaching significance after Bonferroni correction. At 30 min, the median FGF23 was 209% of baseline [95% CI: 154–332%]; at the 4-h nadir, the median was 68% of baseline [95% CI: 54–87%].

We also measured FGF23 in a separate cohort of 18 patients who presented with acute ST-elevation myocardial infarction (STEMI). Table 1 shows the clinical characteristics of these STEMI patients (3 women and 15 men; mean age 70.6 ± 10.18 years). In contrast to the TASH group, the STEMI patients had a moderately reduced left ventricular function at presentation (mean EF 49.5% ± 9.1%). Average renal function remained normal (mean GFR 85.1 ± 34.4 mL/min; serum creatinine 0.97 ± 0.38 mg/dL). Figure 1B depicts the serum FGF23 concentrations in STEMI patients on hospital admission (prior to reperfusion therapy) and again three hours after percutaneous coronary intervention (PCI) recanalization. The median FGF23 on admission was 27.0 RU/mL (15.5–35.75), and this value decreased to 15.5 RU/mL (6.75–34.25) at 3 h post-intervention. This absolute decline in FGF23 was not statistically significant (*p* = 0.0738), despite a medium effect size (r = 0.422). An exploratory analysis of baseline-normalized values showed a median decline to 75.0% of the admission value [95% CI: −54.2% to 4.3%], which reached statistical significance (*p* = 0.0241). However, the wide confidence interval and limited sample size (*n* = 18) with only a single post-baseline time point warrant cautious interpretation. Taken together, STEMI patients showed a trend toward lower FGF23 levels 3 h after reperfusion, consistent with the decline phase observed in the TASH cohort at the 2–4 h window.

## 4. Discussion

Determining the exact onset time of an acute myocardial infarction (AMI) in clinical practice is challenging, making it difficult to track immediate time-dependent changes in circulating biomarkers like FGF23 after the event. Prior studies of FGF23 in cardiac disease have largely measured levels at non-acute time points (e.g., days after infarction or in chronic heart failure), missing the very early phase of acute response. In this context, our study leveraging the TASH model is valuable: TASH closely mimics the pathophysiology of an MI but allows predefined timing of the “infarct” event. This controlled setting enabled us to correlate changes in FGF23 with precise time intervals following myocardial injury, something not feasible in a typical clinical AMI scenario. It must be noted that although TASH involves the induction of a controlled infarct, it does not perfectly replicate the clinical mechanism of a spontaneous STEMI caused by plaque rupture.

In the TASH patients, we observed a rapid surge in FGF23 within 30 min of infarct induction. The approximately twofold rise in such a short span suggests release of FGF23 from pre-formed cellular or tissue stores rather than new protein synthesis. This notion is consistent with biological plausibility, as de novo synthesis would likely take longer than half an hour. Notably, when values were expressed relative to each patient’s baseline, the magnitude of increase in some cases exceeded 100%, underscoring the dynamic nature of this early response. After this initial spike, FGF23 levels declined steadily between 1 and 4 h, indicating clearance of the hormone from circulation. It is worth considering that the initial 30-min FGF23 peak might partly be influenced by the TASH procedure itself (i.e., procedural stress or ethanol injection) rather than the myocardial infarction alone. However, the subsequent clearance phase (1–4 h) is likely a direct consequence of the infarct-triggered release and removal of FGF23 by mechanisms that remain to be clarified. In other words, while the exact cause of the immediate FGF23 surge needs further investigation, the post-spike decline appears to reflect normal hormonal elimination following acute elevation.

By 24 h after TASH, we noted a secondary rise in FGF23 levels, roughly twofold higher than the 4-h. Although this late increase did not reach statistical significance after Bonferroni correction for multiple comparisons (adjusted *p* = 0.14; raw *p* = 0.009), the clear upward trajectory from the 4-h nadir to the 24-h time point is notable and consistent with a biologically meaningful trend. In a pre-specified exploratory sensitivity analysis using normalized values, the increase becomes even more pronounced, further supporting the direction of this effect. We propose that this third phase of FGF23 kinetics (after ~20 h) may be driven by active synthesis and secretion of FGF23 in response to the infarction and the ensuing inflammatory response. In support of this, it is known that after myocardial infarction, immune cells such as neutrophils and macrophages infiltrate the injured myocardium within hours, releasing inflammatory mediators [15]. One of these mediators, oncostatin M (OSM), can stimulate cardiomyocytes to produce and secrete FGF23 within a few hours [6]. It is plausible, therefore, that cardiac cells contribute to the late increase in circulating FGF23 by newly synthesizing the hormone under the influence of OSM and other cytokines. These inflammatory dynamics following TASH (IL-6, CRP) have been documented in comparable patient cohorts from our center [16,17]. This idea aligns with our previous observations in the TASH model: we documented significantly elevated neutrophil counts about 2 h post-TASH and a marked monocytosis by 24 h [17], indicating robust immune activation. We also observed increased circulating interleukin-6 (IL-6) levels at 24 h after TASH [17], which is pertinent because IL-6 is a downstream marker of OSM activity and a general inflammatory response. The parallel rise of IL-6 and FGF23 is consistent with the hypothesis that OSM and the inflammatory cascade are at play, although this association remains correlative and does not establish causality. OSM is a potent inducer of both IL-6 and FGF23 in cardiac myocytes [6]. It is also conceivable that extra-cardiac sources contribute to the 24-h FGF23 surge. OSM can induce FGF23 production in other cell types [6], and prior research suggests osteoblasts in bone might release FGF23 in inflammatory settings [18]. Thus, multiple tissues (heart, bone, and perhaps others) could be collectively responsible for the elevated FGF23 levels observed one day after infarction. Inflammatory markers (CRP, IL-6) and immune cell kinetics following TASH have been characterized previously in comparable patient cohorts from our center [16,17]. The present study was specifically designed to assess FGF23 kinetics. Future studies integrating simultaneous measurements of FGF23, mineral metabolism parameters (phosphate, calcium, 1.25-dihydroxyvitamin D), and inflammatory mediators within the same cohort will be essential to establish causal relationships. Given the strength of the biological rationale and the observed upward trajectory between 4 and 24 h, it is tempting to speculate that circulating FGF23 levels may continue to rise beyond our 24-h observation window, driven by ongoing inflammatory signaling and possible de novo synthesis; longer follow-up studies will be needed to test this hypothesis.

We also examined FGF23 kinetics in real-world STEMI patients to see how they compare with the TASH model. In the STEMI cohort, the trend was a decline in FGF23 about 3 h after reperfusion, which corresponds generally to the early post-infarct period captured in TASH (1–4 h after infarction). The drop in median FGF23 in STEMI was smaller and not significant in absolute terms, which is not surprising given the practical limitations. Unlike the controlled timing in TASH, actual MI patients present at variable times after symptom onset, and it is unlikely that any blood samples were obtained within the first 30–60 min of coronary occlusion. Therefore, if a transient spike in FGF23 occurred shortly after the infarct (as seen at 30 min in TASH), it could have been missed in the STEMI patients by the time of hospital admission. Additionally, patient heterogeneity and the timing of reperfusion introduce variability that can mask subtle changes. When we accounted for this by normalizing to each patient’s baseline, the FGF23 decline at 3 h became statistically significant, in this pre-specified exploratory sensitivity analysis (*p* = 0.0241, effect size r = 0.422), indicating that a modest but consistent drop does occur after reperfusion in most patients. In summary, the STEMI data, though not directly comparable to TASH in terms of precise timing, supports the concept of an early post-infarct decrease in FGF23 levels (following an unobserved initial peak). The magnitude of change in STEMI is dampened, likely due to missing the earliest phase and averaging out of different timelines, but the relative analysis confirms a decrease consistent with the clearance phase seen in TASH.

The triphasic time course of FGF23 levels observed in the TASH model (initial spike, clearance drop, and late recovery toward baseline) is consistent with a dynamic response of FGF23 during the acute phase of myocardial infarction. As our assay does not distinguish between intact FGF23 and its fragments, all mechanistic interpretations of the observed kinetic pattern should be considered hypothesis-generating. It is important to note that FGF23 biology is complex and context-dependent. Intact FGF23 is well known for its hormonal role in regulating phosphate and vitamin D metabolism (acting as a “phosphatonin”) and as a biomarker of chronic kidney disease. However, following acute injury, the effects of FGF23 (and its breakdown fragments) may extend beyond mineral metabolism. FGF23 is secreted as an intact 32 kDa hormone, but it can be proteolytically cleaved into N-terminal and C-terminal fragments that may have distinct or even opposing actions. The C-terminal fragment (cFGF23), for example, can bind FGF23 receptors without requiring the co-receptor Klotho, and it is thought to competitively inhibit the signaling of intact FGF23. According to Goetz et al., excess cFGF23 can prevent the formation of functional FGF23–Klotho–FGFR1c complexes, potentially leading to increases in serum phosphate and calcitriol levels [19]. Paradoxically, another study demonstrated that infusion of a synthetic C-terminal FGF23 fragment in rats increased phosphate excretion and decreased serum vitamin D3 [20], indicating that the fragment’s in vivo effects are not fully understood. In contrast, the N-terminal fragment is often considered inactive since it lacks the receptor-binding C-terminal tail, and little literature exists on any function it might have—it is often treated as a byproduct of FGF23 cleavage.

An intriguing aspect of FGF23’s physiology is the difference between acute versus chronic elevations and the role of its fragments. In our prior work with cultured cardiomyocytes, we found that OSM stimulation led to a large increase in FGF23 production, but only about 3% of the FGF23 released by cardiomyocytes was in the intact, full-length form [10]. The vast majority was cleaved, yielding fragments. This finding suggests that cardiac-derived FGF23 in acute settings is predominantly fragmented, which might serve a regulatory or protective purpose. For instance, it is conceivable that these fragments could counteract the pro-hypertrophic or pro-fibrotic actions of intact FGF23 on the heart (a hypothesis that warrants further investigation). It is possible that a transient burst of intact FGF23 (as we saw at 30 min) could have immediate autocrine or paracrine effects, while the subsequent fragments or declining phase might mitigate those effects or prepare the system for the next phase of repair.

Several limitations of this study warrant discussion. Firstly, and importantly, the C-terminal ELISA used in this study measures total FGF23, encompassing both intact FGF23 and its C-terminal fragments. This assay cannot differentiate biologically active intact FGF23 from inactive or potentially antagonistic fragments. Given the distinct and possibly opposing actions of intact versus cleaved FGF23 discussed above, this represents a major limitation, as the observed kinetic changes may reflect shifts in fragment composition rather than intact hormone alone. Secondly, our sample sizes are modest (TASH *n* = 38, STEMI *n* = 18), which limits the generalizability of the findings and may leave the STEMI cohort underpowered to detect smaller effect sizes (Kendall’s W = 0.518 for TASH; r = 0.422 for the normalized STEMI comparison). Thirdly, medications known to influence FGF23 levels, including phosphate binders and vitamin D supplements, were recorded but not standardized across patients. While the influence of these agents on short-term FGF23 kinetics within 24 h is expected to be limited, a confounding effect cannot be entirely excluded.

Fourthly, this study focused exclusively on FGF23 concentrations and did not include measurements of serum ions (phosphate, calcium), mineral metabolism parameters (1,25-dihydroxyvitamin D), inflammatory markers, or profibrotic mediators. The absence of these parameters limits the ability to assess the functional significance of the observed FGF23 changes and to establish causal relationships with the underlying inflammatory and metabolic milieu.

Fifthly, clinical characterization of the STEMI cohort, including infarct localization, culprit vessel, Killip class, and door-to-balloon time, was incomplete, as these variables were not systematically recorded in the original biomarker study protocol. The small sample size (*n* = 18) further precluded meaningful subgroup analyses. The variable delay between symptom onset and blood sampling in STEMI patients likely contributed to the more heterogeneous and attenuated FGF23 response compared to the precisely timed TASH model. Importantly, the primary finding of this study is the temporal pattern of FGF23 changes rather than the definition of absolute concentration thresholds. While variables such as infarct size and culprit vessel are likely to influence the magnitude of the FGF23 response, they would not be expected to fundamentally alter the directional pattern observed in our data. Future prospective studies with comprehensive clinical phenotyping and larger sample sizes are needed to address this limitation.

Lastly, our observation period was limited to 24 h, and longer follow-up will be needed to determine whether the secondary rise in FGF23 continues beyond this time frame or represents a transient phenomenon. Finally, blinding was partial: the analyst performing the ELISA measurements was aware of the cohort assignment (TASH vs. STEMI) but was blinded to time points and clinical data. Although this is unlikely to affect the objective ELISA results, it should be acknowledged as a methodological limitation.

In conclusion, our study using the TASH model reveals a distinct triphasic time course of circulating FGF23 in the first 24 h after myocardial infarction: an early surge (within minutes), a rapid clearance (over the next few hours), and a later recovery toward baseline with a continuing upward trend beyond the 4-h nadir (by 24 h), possibly reflecting secondary production. These dynamic changes may reflect distinct biological processes at various stages following myocardial injury. The initial surge is consistent with a stress or injury response, the mid-course decline with clearance of acutely released FGF23, and the late recovery with possible involvement of inflammatory signaling and early repair mechanisms. However, these interpretations remain speculative, as the present study measured only total FGF23 concentrations and did not assess inflammatory mediators or repair markers directly. While we cannot entirely rule out procedural effects in the early spike, the overall pattern appears to be a consequence of the infarction and subsequent biological responses. Whether the observed FGF23 dynamics reflect protective or harmful processes, or both, remains an open question. It is conceivable that therapeutic modulation of FGF23 or its fragments during the acute phase of MI could represent a future avenue of investigation, but this will require mechanistic studies employing assays that distinguish intact FGF23 from its fragments, combined with extended follow-up, to establish causal relationships before any therapeutic implications can be considered.

## Figures and Tables

**Table 1:** Baseline Clinical Characteristics of Study Cohorts. Summary of demographic and clinical parameters for 38 patients undergoing therapeutic septal ablation for hypertrophic obstructive cardiomyopathy (TASH) and 18 patients with ST-elevation myocardial infarction (STEMI) who underwent percutaneous coronary intervention. Data include age, sex, left ventricular ejection fraction, co-morbidities, co-medication, glomerular filtration rate, serum creatinine, and other relevant baseline measures.

Number of Patients	38	18
Variables & Groups	TASH	References STEMI
Age in years, mean (SD)	64.2 (±11.7)	70.6 (±10.18)
Female in %	60.5	16.7
LV EF in %, SD	64.2 (±7.4)	49.5 (±9.1)
NYHA, Median	3	2
Hypertension, %	76.3	84
Diabetes Mellitus, %	31.6	21
Family History, %	24.2	21
Atrial fibrillation, %	23.7	0
Hypercholesterolaemia, %	45.7	58
Nicotine, %	16.7	42
CHD, %	20	100
Stroke, %	7.9	5
BMI, kg/m^2^, SD	29.4 (±5.9)	28.33 (±3.8)
**Co-Medication**		
Beta Blocker, %	58	100
ACE-Inhibitor, %	42.1	84
Statins, %	58	100
ASS, %	47.4	95
Aldosterone antagonists, %	8	11
AT1 antagonists, %	13.2	5
Diuretics, %	50	42
**Kidney values**		
GFR, ml/min, SD	77.2 (±20.5)	85.1 (±34.4)
Creatinine, mg/dL, SD	0.9 (±0.2)	0.97 (±0.38)

## Data Availability

The data presented in this study are original and available in the article.
